# Effect of Cyclodextrin Types and Co-Solvent on Solubility of a Poorly Water Soluble Drug

**DOI:** 10.3390/scipharm84040694

**Published:** 2016-10-18

**Authors:** Suporn Charumanee, Siriporn Okonogi, Jakkapan Sirithunyalug, Peter Wolschann, Helmut Viernstein

**Affiliations:** 1 Department of Pharmaceutical Sciences, Faculty of Pharmacy, Chiang Mai University, Chiang Mail 50200, Thailand; siriporn.okonogi@cmu.ac.th (S.O.); jakkapan.s@cmu.ac.th (J.S.); 2 Department of Pharmaceutical Technology and Biopharmaceutics, Faculty of Life Sciences, University of Vienna, Althanstrasse 14, Vienna 1090, Austria; karl.peter.wolschann@univie.ac.at (P.W.); helmut.viernstein@univie.ac.at (H.V.)

**Keywords:** cyclodextrins, co-solvent, co-solubilization, inclusion complexation, piroxicam

## Abstract

The aim of the study was to investigate the solubility of piroxicam (Prx) depending on the inclusion complexation with various cyclodextrins (CDs) and on ethanol as a co-solvent. The phase-solubility method was applied to determine drug solubility in binary and ternary systems. The results showed that in systems consisting of the drug dissolved in ethanol–water mixtures, the drug solubility increased exponentially with a rising concentration of ethanol. The phase solubility measurements of the drug in aqueous solutions of CDs, β-CD and γ-CD exhibited diagrams of A**_L_**-type, whereas 2,6-dimethyl-β-CD revealed A**_P_**-type. The destabilizing effect of ethanol as a co-solvent was observed for all complexes regardless of the CD type, as a consequence of it the lowering of the complex formation constants. In systems with a higher concentration of ethanol, the drug solubility was increased in opposition to the decreasing complex formation constants. According to this study, the type of CDs played a more important role on the solubility of Prx, and the use of ethanol as a co-solvent exhibited no synergistic effect on the improvement of Prx solubility. The Prx solubility was increased again due to the better solubility in ethanol.

## 1. Introduction

The solubility of a drug is an essential property required to achieve a sufficient bioavailability. It is important in all steps of drug product development, from drug discovery and dosage forms development to clinical applications. Many drugs, particularly those recently discovered, present a large and complex molecular structure which leads, in some cases, to a poor water solubility. Various methods, for example, salt formation, co-solvency, micellar solubilization, solid dispersions and complexation by cyclodextrins (CDs), nanosizing and particles engineering have been used successfully to increase the solubility of drugs [1,2,3,4]. Recently, lipid drug delivery systems or nano-emulsions have been investigated, especially for highly lipophilic drugs [5,6]. Although these conventional solubilization techniques have been well-described and used successfully for many drugs, each possesses limitations in terms of the solubilizing capacity, patient acceptability and safety [7,8]. Two or more solubilization techniques are then combined, aiming to obtain the synergistic solubilizing effect and/or minimize, to some extent, its drawbacks [9,10,11].

The combined method of CDs complexation and the use of a co-solvent is an approach of interest which has been reported by a number of studies [12,13,14,15,16]. In theory, only the molecular structure of the guest molecule is responsible for the inclusion complex formation with CDs; the presence of a co-solvent, thus, helps by facilitating the complex formation by dissolving the guest before entering the cavity. In addition, even more importantly, the co-solvent could also dissolve the excess guest molecules which are not incorporated into the CDs cavity [17]. The synergistic effect on improving the drug solubility is, in many cases, promisingly achieved by the co-existance of the co-solvent and CDs.

However, a destabilizing effect on CDs complexation, caused by co-solvents, has also been reported [18]. Two mechanisms have been discussed: firstly, the co-solvent can influence the polarity of the medium [19]. At a certain concentration of the co-solvent, the polarity may be lowered to a level that is suitable for a drug molecule to be favorably solvated. This decreases the driving force of the drug molecule to enter the CDs cavity. Secondly, the co-solvent can compete with a drug molecule to occupy the space in the CDs cavity [20,21,22]. Studies involving a co-solvent and CDs on the solubility improvement of a drug are still controversial. The presence of a co-solvent and CDs can provide either a synergistic or antagonistic effect on drug solubility; depending on whether the promoting or destabilizing effect is outweighed.

In recent years, a number of CD derivatives have been introduced such as hydroxypropyl-β-cyclodextrin, dimethyl-β-cyclodextrin and sulfobutylether-β-cyclodextrin, aiming to improve the water solubility of drugs and to expand the area of application [19].

One of the interesting applications of CD complexation is its ability to separate the enantiomers of a drug [23]. As mentioned in the literature, we found that only β-CD and hydroxypropyl-β-cyclodextrin (HP-β-CD) have been used widely as hosts for studying the effect of co-solubilization by co-solvents. In general, the physicochemical properties and complexation ability of the CD derivatives are different from the native ones. The influence of the CD type, together with the influence of co-solvents on the CDs complexation, has not been reported extensively up to now.

In the present study, we investigated the effect of the CD type and ethanol, as a co-solvent, on the complexation affinity of CDs and piroxicam. The CDs used were two natural CDs: β-CD (BCD) and γ-CD (GCD); and 2,6-dimethylated-β-CD (MeBCD). The effect of the CD type and ethanol was evaluated as a function of drug solubility and the complexation efficiency (CE).

**Figure 1:**
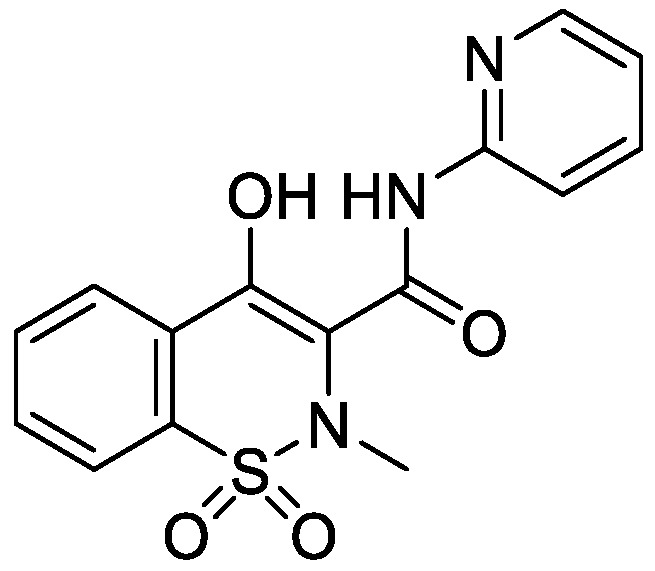
Structure of piroxicam.

Piroxicam (Prx), (4-hydroxy-2-metyl-*N*-(2-pyridyl)-2*H*-1,2-benzothiazine-3-carboxamide-1,1dioxide)—a non-steroidal anti-inflammatory drug—was used as a model compound in this study. Prx is categorized under the Biopharmaceutical Classification System, (BCS) Class II; its structure is shown in Figure 1.

## 2. Materials and Methods

### 2.1. Chemicals

Piroxicam (Prx), the model drug, was purchased from Sigma-Aldrich (St.Louis, MO, USA). The cyclodextrins used, namely β-cyclodextin (BCD), γ-cyclodextrin (GCD) and methylated-β-cyclodextrin (MeBCD) were from Wacker-Chemie, Munich Germany. Ethanol was analytical reagent grade.

### 2.2. The Calibration Curve Construction

The Prx solutions of exact concentrations of 3–15 μg/mL were prepared using ethanol. The absorbance of the solutions was measured by using an ultraviolet (UV)-spectrophotometer (UV-2450, Shimadzu, Kyoto, Japan) at a maximum wavelength of 359 nm. Each experiment was performed in triplicate. The calibration curve was drawn by plotting the absorbance against the known concentrations. The linear regression analysis was applied to construct the calibration curve.

### 2.3. Determination of the Drug Solubility in the Co-Solvent–Water Binary System

The solubility of Prx was measured as a function of ethanol concentration up to 20%. Briefly, an excess amount of Prx was accurately weighed and placed into the glass screwed cap bottle. The appropriate volume of ethanol and water was added to obtain varying percentages of ethanol: 1%, 3%, 5%, 7%, 10% and 20% by volume respectively. The bottles were sonicated using ultrasonic cleaner (Elma, Singen, Germany) of 50 kHz for a few minutes to ensure homogeneity of the mixtures and then placed on the magnetic plate (Variomag^®^, Thermo Fisher Scientific, Waltham, MA, USA). The mixture was stirred using a small magnetic bar at 150 rpm for 48 h in a thermostatic incubator with temperature controlled at 25 ± 0.1 °C. The mixture was then passed through a 0.45 μm membrane filter to obtain a clear filtrate. The filtrate was diluted if necessary and the absorbance was measured at 359 nm using a UV-spectrophotometer (UV-2450). The absorbance was converted into Prx concentrations using the calibration curve. The influence of ethanol on Prx solubility was assessed by plotting the concentration of Prx against the percentage of ethanol. The results were obtained using Yalkowsky and Roseman’s logarithmic—linear model [24].

### 2.4. Phase Solubility Study of the Prx–CDs Binary System

The phase solubility study was conducted according to the method previously described [25]. An excess amount of Prx was placed in the aqueous solution of different CDs, namely, BCD, GCD and MeBCD. The concentrations of BCD and GCD were 0, 3, 6, 9 and 12 mM whereas those of MeBCD were 0, 30, 60, 90 and 120 mM. The low concentration range of the native CDs was used according to the low water solubility. The mixtures were magnetically stirred at 150 rpm during equilibration in the controlled temperature cabinet at 25 ± 0.1 °C for 48 h. At the end of the equilibrium time, the absorbance of the filtrate in each bottle was measured using a UV-spectrophotometer (UV-2450) at a maximum wavelength of 359 nm against the solution of the CD as a blank. The Prx content was calculated using a calibration curve. The phase solubility profiles of each CD were established.

### 2.5. Phase Solubility Study of the Prx–CDs–Ethanol Ternary System

The procedures resembled the phase solubility of the binary system as previously described. Each prepared ternary system composed of the excess amount of Prx and ethanol of varying concentrations of 0, 1%, 3%, 5%, 7%, 10% and 20%; the various CDs concentrations of 0, 3, 6, 9, 12 mM for BCD and GCD; and 0, 30, 60, 90, 120 mM for MeBCD. Each mixture was equilibrated at the temperature 25 ± 0.1 °C. They were stirred continuously at 150 rpm, while being kept for 48 h to ensure the equilibrium. The soluble Prx content in each system was analysed using a UV-spectrophotometer (UV-2450) at a maximum wavelength of 359 nm against the CD/ethanol solution as a blank and by applying the calibration curve. The simultaneous effects of ethanol and CDs on the Prx solubility were illustrated by histogram plots.

## 3. Results and Discussion

The calibration curve or Prx was constructed using UV-spectrophotometer (UV-2450) at a maximum wavelength of 359 nm. Ethanol had no effect on the UV absorption of Prx at this wavelength [26]. The following equation was obtained and it was used for determining the concentration of Prx in all investigated solutions:

y = 0.0485x − 0.0168   R^2^ = 0.9996

**Figure 2:**
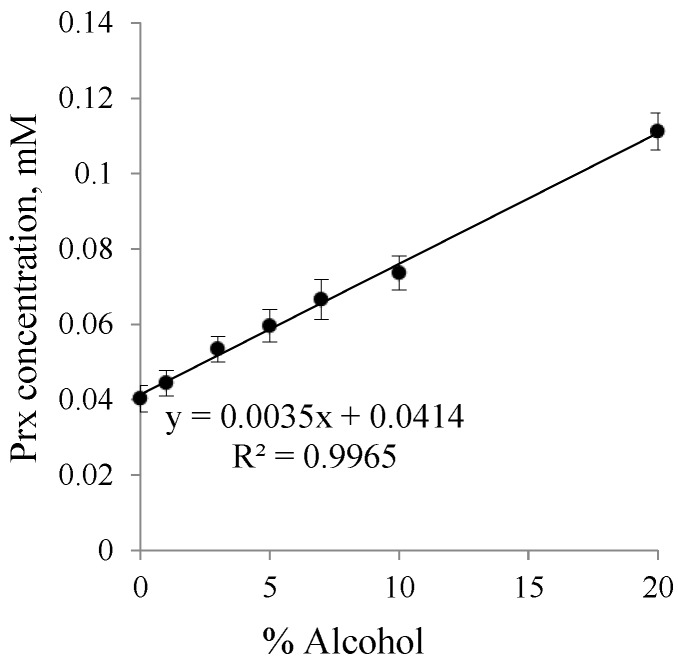
Solubility profile of piroxicam (Prx) in an ethanol–water system.

Figure 2 shows a linear relationship between Prx concentration and the percentage of ethanol. The y-intercept, which indicates the intrinsic solubility of Prx in water, is 0.0403 mM (equivalent to 13.4 μg/mL) at 25 °C. The value was slightly higher than that previously reported by Yazdanian et al. [27], which was 12.0 μg/mL in simulating fed intestinal fluid at pH 5.0 at the same temperature. This discrepancy results from the different pH value of the medium. In our study, the pH of the Prx filtrate was about 6.7 ± 0.15. At this pH, the predominantly form of Prx is the enolic-ionized form, which helps to increase the solubility of Prx. Organic modifiers, such as acetonitrile or ethanol, could influence the shift of the pKa value [28,29]. In an ethanol–water system, the increase in Prx solubility was enhanced by the pH effect and the presence of ethanol, acting as a co-solvent and a pKa modifier, respectively.

**Figure 3:**
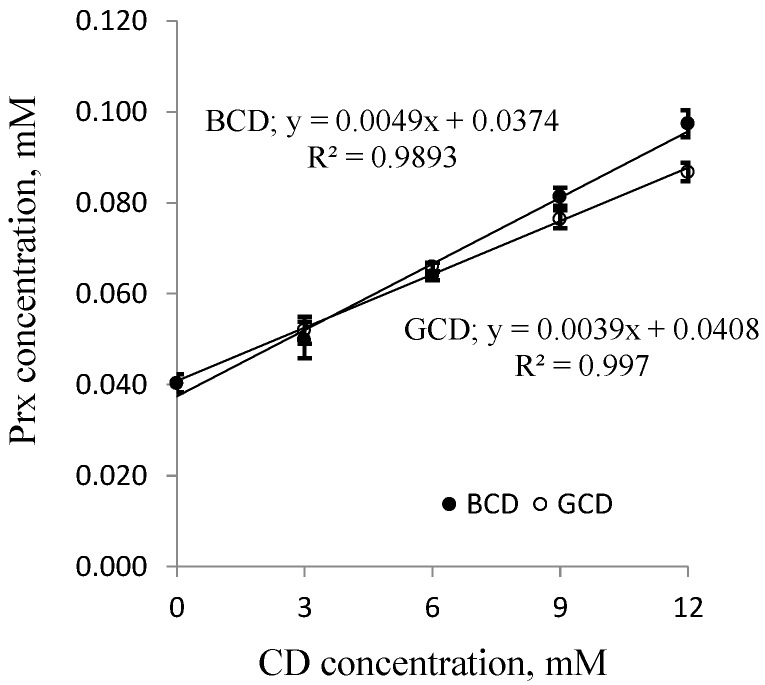
Phase solubility diagrams of Prx-CD inclusion complexes; **●** β-CD (BCD) and ○ γ-CD (GCD). CD: cyclodextrin.

**Figure 4:**
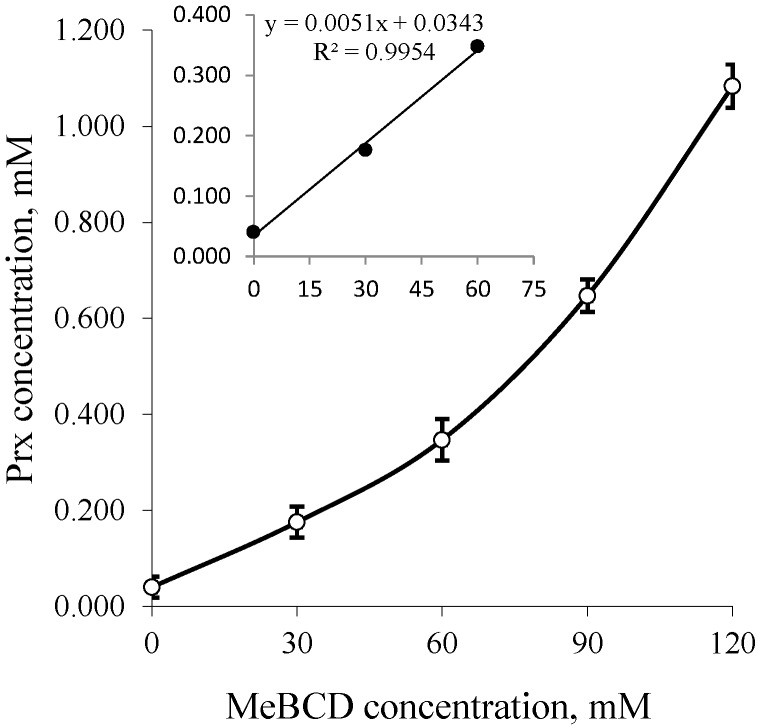
Phase solubility diagrams of Prx–MeBCD inclusion complexes; Insert: Up to 0–60 mM of MeBCD. MeBCD: 2,6-dimethylated-β-CD.

The solubility of Prx in the binary system containing different types of CDs with various concentrations is illustrated in the phase solubility diagrams, in Figure 3 for BCD and GCD, and in Figure 4 for MeBCD.

For BCD and GCD, a solubility diagram of the A**_L_**-type was obtained, signifying that the inclusion complex of Prx and either of the two native CDs consisted of a 1:1 molar ratio. The results are in agreement with previous reports [13,21]. From this linear relationship, the complex formation constants, k_1:1_, can be calculated from the y-intercept and slope of the straight line according to the following equations:

(1) $$\left[D\right]+\left[CD\right]\;\overset{k1:1}{↔}\;\left[D-CD\right]$$

(2) $$k_{1:1}\;=\frac{Slope}{S_{0}\left(1-Slope\right)}\;\;$$

where S_0_ is the intrinsic solubility of Prx in water.

The complexation efficiency can be calculated from Equation (3).

CE = S_0_ × k_1:1_ (3)

In the case of MeBCD, the phase solubility curve was positively deviated from linearity. It was assigned as an A**_P_**-type. This indicates the formation of higher order inclusion complexes. The complex formed may be second order or more with respect to MeBCD concentrations. However, at a lower concentration of up to 60 mM, as illustrated in the insert of Figure 4, a linear phase-solubility curve was obtained. The first part of the plot was used to calculate the complex formation constant k_1:1_, which can be compared to those of the other CDs. The k_1:1_ and CE values of all complexes are summarized in Table 1.

The S_0_ values obtained from the phase-solubility diagrams agreed with the directly determined value, which was 0.0403 mM. The k_1:1_ value of the Prx–BCD complex was comparable to that previously reported, which was 103.5 M^−1^ [9]. The inclusion complex of Prx–BCD was somewhat more stable than Prx–GCD. A smaller cavity space of BCD may be more convenient for the molecular size of Prx. Significantly, MeBCD forms a more stable complex than the two natural CDs, which is demonstrated by the higher k_1:1_ value and the related CE value. CE is a useful parameter to select suitable CDs for the complex formulation. Up to now, MeBCD has not been accepted as generally recognized as safe (GRAS) ingredient, therefore, only the formulations containing the Prx–BCD inclusion complex are available on the market.

The effect of ethanol on the Prx–CDs complexation was measured using phase-solubility studies. The k_1:1_ values were estimated and they are summarized in Table 2. It was shown that ethanol lowered the k_1:1_ value regardless of the CD type. This observation indicates that the complexation is destabilized by the presence of ethanol. This is in agreement with many reports as previously described.

**Figure 5:**
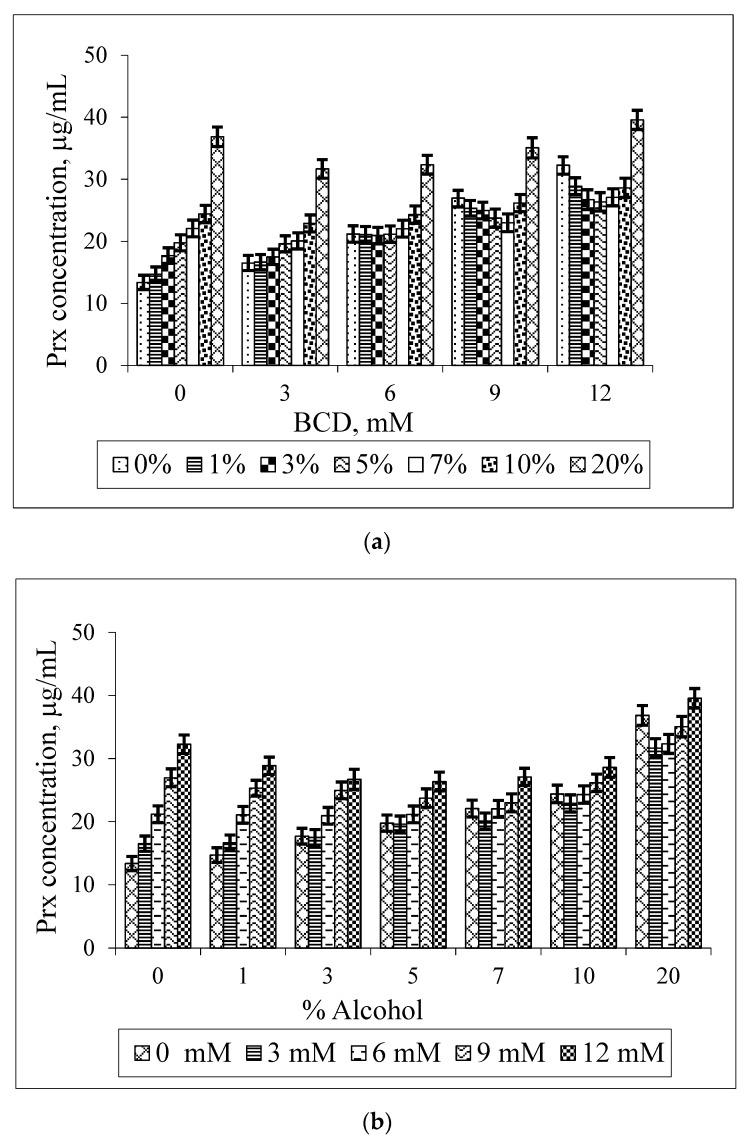
Effect of BCD concentrations on the solubility of Prx in Prx–BCD–ethanol ternary mixtures, varying the percentage of ethanol (**a**); Effect of ethanol on the solubility of Prx in Prx–BCD–ethanol ternary mixtures varying BCD concentrations (**b**).

Figure 5 illustrates the bilateral effect of BCD and ethanol on the Prx solubility. It is shown that the Prx solubility increases linearly in the binary system (when either BCD or ethanol is absent). However, as shown in Figure 5a, the increase in the percentage of ethanol does not enhance the drug solubility at every BCD concentration. This synergistic effect, the increase in the Prx solubility upon rising the ethanol percentage, is only observed at low concentrations of BCD i.e., 3 mM. At the slightly higher BCD concentration of 6 mM, instead of being further increased, the drug solubility is unaltered by the increase in ethanol percentage up to 7%. Two explanations for this co-solvent effect can be given: firstly, ethanol is less polar than water; it can compete with the drug to occupy the BCD’s cavity. This was supported by the molecular dynamics study demonstrating the interaction between BCD and ethanol [21,22]. Instead of dissolving the non-complexed drug only, the ethanol molecule forms an inclusion complex with BCD. Secondly, BCD has a relatively low water solubility of 1.85 g/100 mL, which is equivalent to 16.3 mM and is practically insoluble in ethanol [24]. Both effects led to the destabilization of Prx–BCD complexes. This is confirmed by the decrease in the k_1:1_ values, as shown in Table 2. Thus, no change of the drug solubility is due to a balance between the promoting and destabilizing effect of both BCD and ethanol.

According to the solubility limitation of BCD, this effect should be more pronounced in systems containing a higher concentration of BCD (9 mM, 12 mM) and at a higher percentage of ethanol (10%–20%). Surprisingly, the opposite result was observed. At a higher concentration, ethanol could reduce the polarity of the solution, thus providing an optimal microenvironment for Prx to be sufficiently solvated. This effect diminished the driving force of the complex formation. At this condition, although the drug solubility by complexation is decreased, the overall solubility of the drug is improved due to the solubilizing effect of ethanol as a co-solvent. In other words, the solubility enhancement by the co-solvent is compensating the effect of complexation. As shown in Figure 2, the co-solvent enhances the drug solubility in exponential terms, whereas the CDs increase the drug solubility more or less linearly (Figure 3 and Figure 4).

The increase in drug solubility, compensated by the co-solvent effect, resulted in a non-linear solubility curve. The U-shaped solubility curve with the minima region is depicted in Figure 5b. In this study, the minima region is clearly shown at a higher BCD concentration (9 mM, 12 mM) at 3%–7% ethanol. The solubility curve with a minima region is in coincidence with those previously reported [12,17].

**Figure 6:**
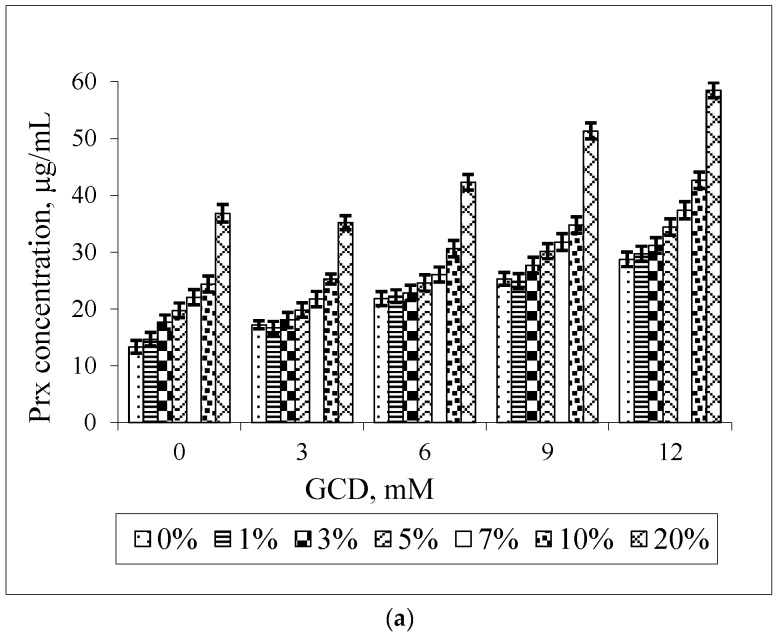

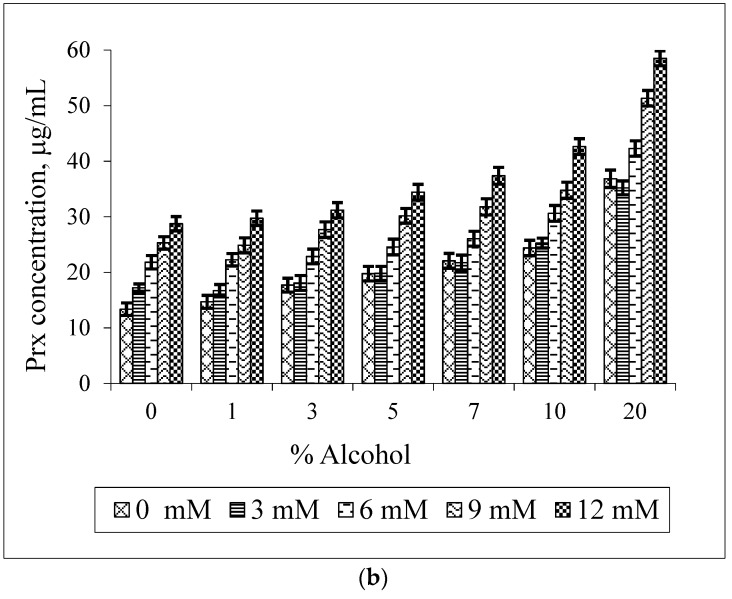
Effect of GCD concentrations on the solubility of Prx in Prx–GCD–ethanol ternary mixtures varying the percentage of ethanol (**a**); effect of ethanol on the solubility of Prx in Prx–GCD–ethanol ternary mixtures varying GCD concentrations (**b**).

Figure 6 shows the solubilizing effect of GCD and ethanol on the Prx solubility. It can be noted that the solubility of Prx is increased in all cases. This is not in agreement with the decrease in the K_1:1_ value, as presented in Table 2. However, the K_1:1_ value of the Prx–GCD complexes is slightly decreased upon increasing the percentage of ethanol. It seems that the stability of the Prx–GCD complex is somewhat less sensitive to ethanol. Compared to BCD, GCD has a higher water solubility (23.2 g/100 mL or 168 mM) which is far from the maximum concentration used in the study (12 mM). Moreover, it is slightly soluble in ethanol (0.1 g/100 mL). In the presence of ethanol, the marked decrease in GCD solubility was not expected. However, from the lowering of the K_1:1_ value, the enhancement of Prx solubility should be mainly due to the solubilizing effect of ethanol instead of complexation.

**Figure 7:**
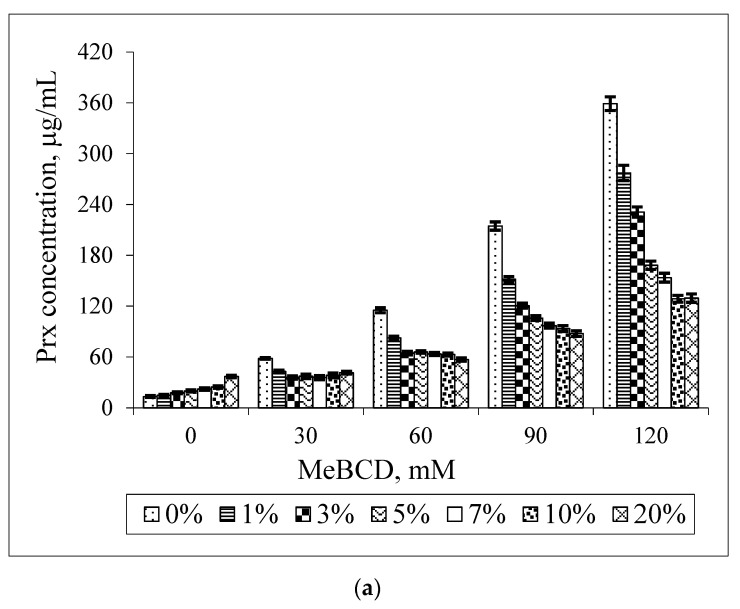

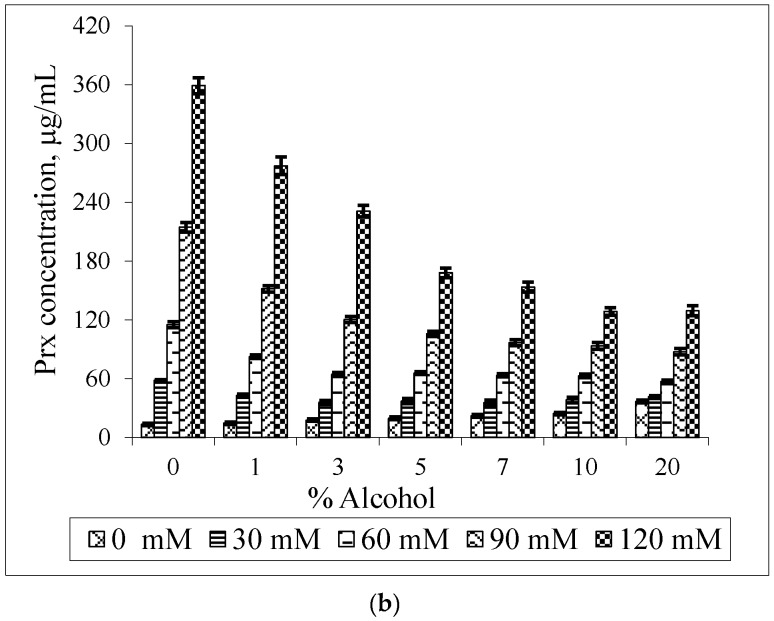
Effect of MeBCD concentrations on the solubility of Prx in Prx–MeBCD–ethanol ternary mixtures varying the percentage of ethanol (**a**); effect of ethanol on the solubility of Prx in Prx–MeBCD–ethanol ternary mixtures varying MeBCD concentrations (**b**).

**Figure 8:**
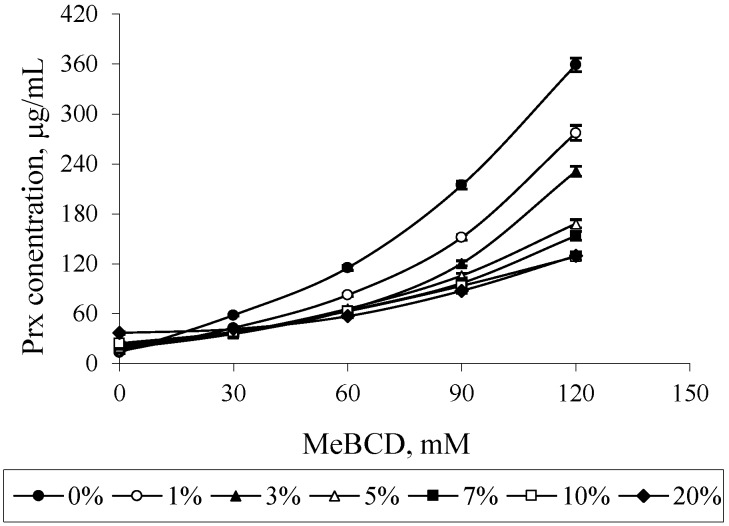
Effect of ethanol on the solubility of Prx in Prx–MeBCD–ethanol ternary mixtures varying MeBCD concentrations.

The effect of MeBCD and ethanol on the drug solubility was illustrated in Figure 7. The presence of ethanol had no effect on MeBCD solubility in the ternary mixture, since it is soluble in water (>50 g/100 mL) and also in ethanol. The solubility of Prx was decreased in the presence of ethanol and MeBCD. It was more pronounced at a higher concentration of MeBCD (90 mM and 120 mM) and the higher percentage of ethanol 5%–20%. Figure 8 clearly demonstrated that the drug solubility decreased when increasing the ethanol percentage.

In the case of MeBCD, it was clearly shown that the complex formation plays an important role in the solubility enhancement of Prx compared to the effect of the co-solvent. The methylation of natural BCD at OH (2) and OH (6) is known to improve the complexation efficiency of MeBCD. Due to the high complexation efficiency, the decrease in Prx solubility might be anticipated by the competition of ethanol in the MeBCD cavity. However, the effect of ethanol on lowering the polarity of the medium may also play a role to some extent, particularly at higher percentages of ethanol.

## 4. Conclusions

In general, various CD derivatives and the co-solvent can affect the drug solubility in different ways: increasing, decreasing or even keeping it unchanged. The causes of these observations can be rationalized by many reasons. In this study, we investigated the effect of the CD type and ethanol on the solubility of piroxicam using phase-solubility studies. From our results, we conclude that the concentrations of both CDs and ethanol are primary factors. More importantly, we emphasize that the solubility and the complexation ability of the CDs are key factors for these effects. In other words, the type of CD plays important roles on the observations. The limited solubility of BCD in both water and ethanol disallows its application in CD-co-solvent combined systems for medicinal applications. Although, an increase in the piroxicam solubility in the systems containing ethanol of 10% or higher was observed, it is not acceptable for applications. Similarly, ethanol affects GCD complexation ability and the improvement of the drug solubility is attained only at a higher percentage of ethanol. For MeBCD, which is highly soluble in water and ethanol and possesses high complexation ability, the destabilizing effect of ethanol on the MeBCD–Prx complex is remarkable and results in a decrease in the drug solubility.

## Figures and Tables

**Table 1:** The intrinsic solubility of piroxicam (Prx), the complex formation constants and the complexation efficiency of Prx–CDs complexes.

CDs	S_0_ (mM)	k_1:1_ (M^−1^)	CE × 10^−3^ (= S_0_ × k_1:1_)
BCD	0.0374	132	4.9
GBD	0.0408	96	3.9
MeBCD	0.0343	149	5.1

CD: cyclodextrin; BCD: β-CD; GBD: γ-CD; MeBCD: 2,6-dimethylated-β-CD; S_0_: intrinsic solubility of Prx in water; k_1:1_: complex formation constant; CE: complexation efficiency.

**Table 2:** Complex formation constants of Prx–CDs in the ternary systems.

% Alcohol	k_1:1_ (M^−1^)		
	BCD	GCD	MeBCD
0	132	96	149
1	88	90	88
3	52	76	50
5	28	75	43
7	**	69	35
10	**	69	28
20	**	61	9.5

** Cannot be calculated as there are deviations from linearity.
